# Cutaneous Manifestations of “Lupus”: Systemic Lupus Erythematosus and Beyond

**DOI:** 10.1155/2021/6610509

**Published:** 2021-05-18

**Authors:** Elizabeth E. Cooper, Catherine E. Pisano, Samantha C. Shapiro

**Affiliations:** ^1^Department of Dermatology, Dell Medical School at the University of Texas, Austin 78701, USA; ^2^Department of Medicine, Division of Rheumatology, Dell Medical School at the University of Texas, Austin 78701, USA

## Abstract

Lupus, Latin for “wolf,” is a term used to describe many dermatologic conditions, some of which are related to underlying systemic lupus erythematosus, while others are distinct disease processes. Cutaneous lupus erythematosus includes a wide array of visible skin manifestations and can progress to systemic lupus erythematosus in some cases. Cutaneous lupus can be subdivided into three main categories: acute cutaneous lupus erythematosus, subacute cutaneous lupus erythematosus, and chronic cutaneous lupus erythematosus. Physical exam, laboratory studies, and histopathology enable differentiation of cutaneous lupus subtypes. This differentiation is paramount as the subtype of cutaneous lupus informs upon treatment, disease monitoring, and prognostication. This review outlines the different cutaneous manifestations of lupus erythematosus and provides an update on both topical and systemic treatment options for these patients. Other conditions that utilize the term “lupus” but are not cutaneous lupus erythematosus are also discussed.

## 1. Introduction

Skin involvement is often a prominent feature of systemic lupus erythematosus (SLE), a multiorgan, chronic autoimmune disorder that can lead to disability and death [1, 2]. The strongest risk factor for SLE is gender, with a female-to-male incidence ratio of 7 to 15 : 1 in adults and 3 to 4 : 1 in children [3]. Though there is a less dramatic gender predominance in patients who have isolated cutaneous lesions, the female-to-male ratio in these patients is still 3 : 1 [1]. It should be noted that SLE is one of the top 20 leading causes of death in females between 5 and 64 years of age [2]. SLE is four times more prevalent in black women than in white women, and patients of African descent tend to develop disease earlier and have higher mortality [3–5].

While SLE commonly has cutaneous manifestations, cutaneous lupus may occur in the absence of systemic lupus erythematous. Acute cutaneous lupus erythematosus (ACLE), subacute cutaneous lupus (SCLE), and discoid lupus (DLE) are the three most common manifestations of cutaneous lupus erythematosus (CLE). There are also several less common cutaneous manifestations of lupus, including lupus tumidus, lupus panniculitis, bullous SLE, the toxic epidermal necrolysis variant of lupus, chilblain lupus, hypertrophic or verrucous discoid lupus, mucosal discoid lupus, and lichenoid cutaneous lupus-lichen planus overlap syndrome [6, 7]. Complicating the picture, several skin conditions that are separate and distinct from lupus erythematosus make use of the word “lupus” as well: lupus vulgaris, lupus miliaris disseminatus faciei, and lupus pernio. These skin conditions are related to tuberculosis, granulomatous rosacea, and sarcoidosis, respectively. These are not true forms of CLE.

**Figure 1:**
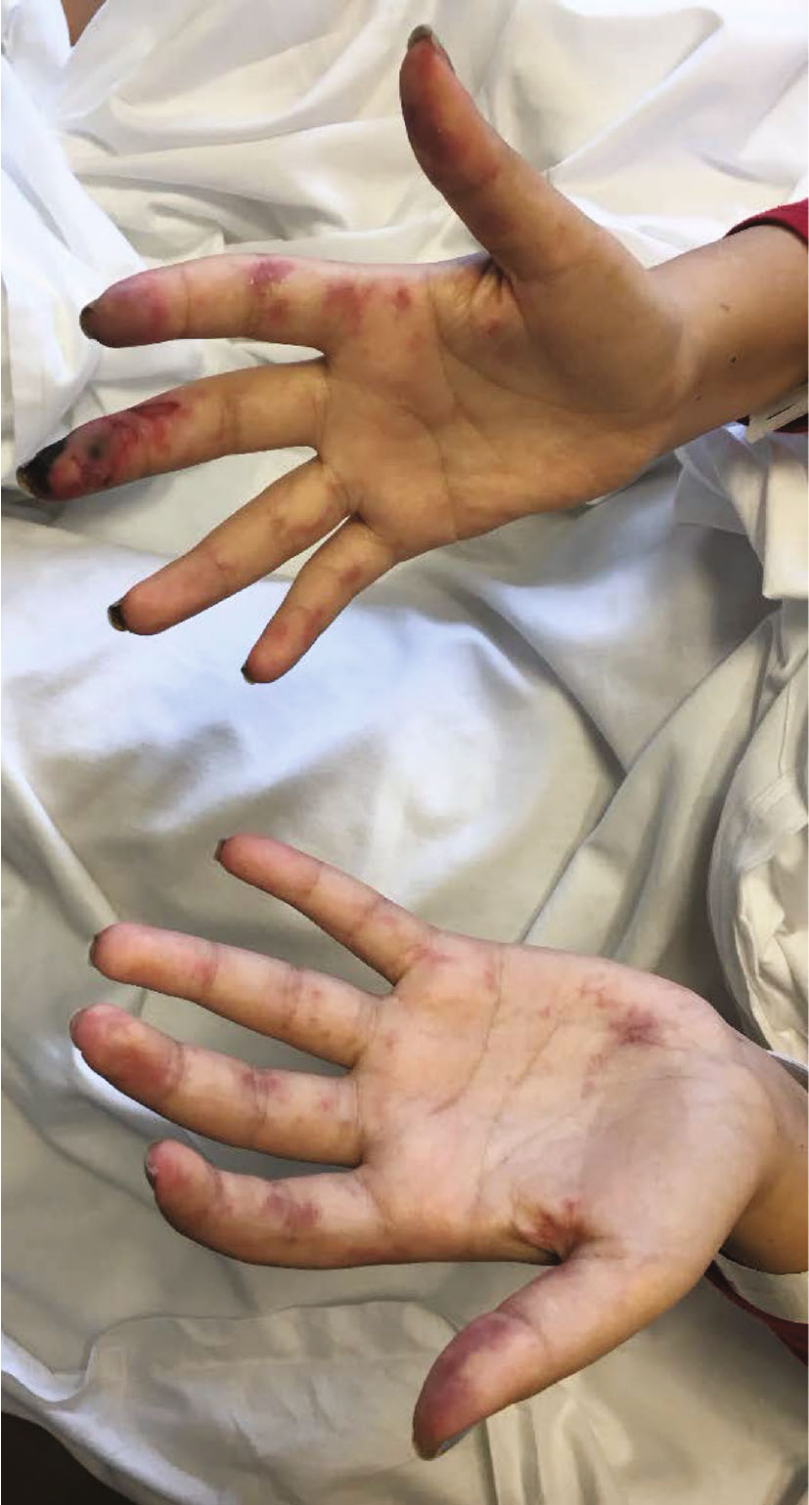
Lupus vasculitis, with digital ischemia and mesenteric ischemia, ultimately resulting in perforated bowel.

After initial diagnosis of CLE, risk of progression to SLE is between 5 and 18% within three to five years [8–10]. Approximately one-third of CLE patients have an existing diagnosis or will be diagnosed with SLE in the future [8]. Patients with ACLE, bullous lupus, and nonspecific cutaneous lesions of lupus (e.g., vasculopathic lesions, see Figure 1) all have a higher risk of developing systemic lupus when compared to individuals diagnosed with SCLE, DLE, lupus tumidus, lupus panniculitis, or chilblain lupus [1].

On a molecular level, most variants of CLE are characterized by a lichenoid tissue reaction as a result of keratinocyte, endothelial cell, and dendritic cell activation [1]. Production of type I interferons with subsequent cluster of differentiation 4+ (CD4+) and CD8+ T cell recruitment and activation leads to cytotoxic keratinocyte damage [1]. CLE results from a complex interplay of genetic and environmental factors [1, 3]. Ultraviolet radiation, certain medications, smoking, and viral infection can trigger an inflammatory cascade involving cells of the skin and recruited inflammatory cells [1, 3, 11–13]. Genetic variation based upon parentage and gene mutations contributes to the wide variation in clinical presentation of cutaneous LE. The three major types of CLE are not mutually exclusive, and more than one type of cutaneous lesion may occur in a single patient [6, 7]. The goal of this review is to critically evaluate the most recent literature on lupus erythematosus-specific cutaneous disease, as well as address cutaneous findings of unrelated conditions that make use of the term “lupus” as a descriptor.

## 2. Cutaneous Manifestations of Lupus

### 2.1. Acute Cutaneous Lupus Erythematosus

Acute cutaneous lupus erythematosus (ACLE) is frequently associated with systemic lupus erythematosus, and it exists in both localized and generalized forms. 95% of patients with ACLE have a positive antinuclear antibody (ANA). In both subtypes of ACLE, flare-ups of rash frequently parallel systemic disease activity, though exceptions may occur. ACLE lesions typically resolve without scarring, though postinflammatory dyschromia may occur, especially in darker skinned individuals [6, 7, 14].

**Figure 2:**
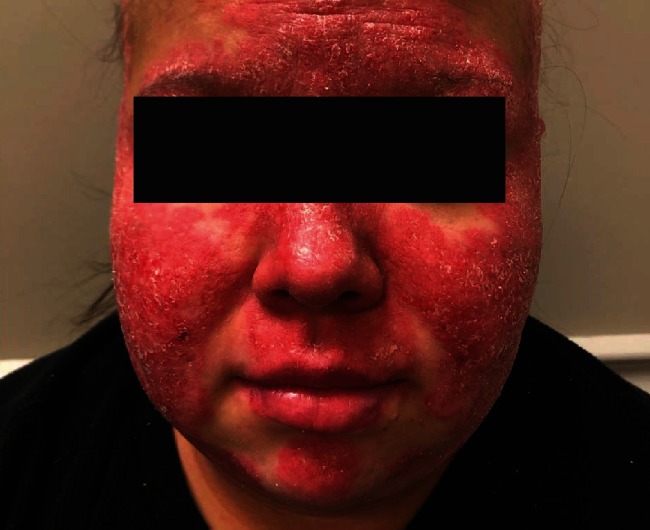
Acute cutaneous lupus erythematosus. Classic malar “butterfly” rash of the forehead, chin, and malar cheeks. Note the relative sparing of the nasolabial folds.

The localized form of ACLE is commonly described as a malar or “butterfly” rash that covers the cheeks and nasal bridge (Figure 2). The forehead and anterior neck may be included, but the nasolabial folds are spared. Confluent, reddish-purple discoloration with mild edema and/or papules is common. The rash classically lasts days to weeks and can be triggered by sun exposure. It is present at diagnosis in 40-52% of SLE patients [15]. The rash may commonly be mistaken for rosacea, which presents with papules and pustules, or seborrheic dermatitis, which involves the nasolabial folds [7]. Telangiectasias, erosions, dyspigmentation, and poikiloderma are all clues that support a diagnosis of ACLE. A malar rash may also occur in dermatomyositis and can be difficult to differentiate from the classic malar rash of ACLE. However, the malar rash of dermatomyositis classically does not spare the nasolabial folds [16].

The generalized form of ACLE is commonly described as a maculopapular rash, or photosensitive dermatitis. It is less common than localized ACLE. The rash may occur both above and below the neck, with a predilection for sun-exposed areas. This form of ACLE may again resemble skin findings seen in dermatomyositis, though the rash involving the hands in ACLE has been described as “reverse Gottron's,” as it involves the skin located between the finger joints, and not the skin overlying the joints [6].

Skin biopsy in ACLE reveals basal layer degeneration, edema of the upper dermis, interface dermatitis with a mononuclear cell infiltrate at the dermal-epidermal junction, mucin deposition, hyperkeratosis, and perivascular and periadnexal inflammation (lymphocytic infiltrate) [1]. Direct immunofluorescence (DIF) can demonstrate the “lupus band” in a majority of cases, which refers to a granular deposition of immunoglobulins and complement at the dermal-epidermal junction [17]. While a positive lupus band test supports a diagnosis of ACLE, a negative DIF does not rule it out [6, 7]. Immunoglobulin M (IgM) and complement 3 (C3) are most commonly detected, and the presence of additional immunoreactants (i.e., IgG, IgA, C1q, and C4), an uninterrupted linear band, and increased intensity of staining all increase the specificity and predictive value of this finding. DIF can be useful for distinguishing cutaneous lupus from other inflammatory skin conditions [18, 19]. Many studies have shown that SLE patients will frequently have positive DIF when sun-protected, nonlesional (i.e., no rash) skin is biopsied [20]. Of note, a nonlesional lupus band test can be positive in patients with other autoimmune diseases [18, 19, 21, 22]. It is important to note that histopathology with DIF cannot differentiate between the rashes of lupus and dermatomyositis, as both may have similar findings [18, 21].

First-line treatment of ACLE includes general preventative measures such as sun protection and smoking cessation (see Table 1) [6, 7]. Local treatments include topical steroids or calcineurin inhibitors, especially in mild cases [6, 7, 23]. Antimalarial agents such as hydroxychloroquine and chloroquine are recommended as first-line systemic agents. Dramatic clinical improvement after administration of these drugs in ACLE has been repeatedly demonstrated in the literature, including one recent meta-analysis which established significant response in 91% of ACLE cases [23]. Short courses of oral corticosteroids may be required in severe or refractory cases, specifically during a flare-up when bridging to slower-acting steroid-sparing medications. However, chronic systemic steroid use is to be avoided due to multiple adverse effects [6].

### 2.2. Toxic Epidermal Necrolysis (TEN) Variant of Lupus

The TEN variant of lupus is a subtype of ACLE that presents as large areas of erythema and denuded skin, similar to the severe cutaneous adverse reactions of Stevens-Johnson syndrome (SJS) and toxic epidermal necrolysis (TEN). The TEN variant of lupus can be seen in both CLE and SLE. The rash may evolve subacutely from more typical lupus rashes over time (e.g., subacute cutaneous LE, or from the photosensitive maculopapular rash of ACLE), or it may develop more rapidly *de novo* [1, 24, 25].

It can be difficult to distinguish SJS/TEN from the TEN variant of lupus. Patients with lupus may have SJS/TEN in isolation, drug-induced SJS/TEN with “concomitant aggravation of lupus erythematosus,” or the TEN variant of lupus [26]. When attempting to differentiate etiologies, a careful history with attention paid to offending drug agents is paramount. It is helpful to confirm a prior diagnosis of CLE or SLE and assess for a preceding lupus flare. Sparing of the mucous membranes, or minimal/focal involvement of the mucous membranes, and evident photodistribution of rash may favor the diagnosis of lupus clinically.

Histopathology can also be helpful in identifying lupus rashes. In typical SJS/TEN, multiple necrotic keratinocytes are present within the entire epidermis; vacuolar changes and lymphocytic infiltrate are typically absent or sparse. In contrast, solitary necrotic keratinocytes in the lower epidermis, junctional vacuolar changes, dense periadnexal and perivascular lymphocytic infiltrate, and mucin argue for the TEN variant of cutaneous lupus [25].

The most commonly used treatment for the TEN variant of ACLE is systemic corticosteroids, with either hydroxychloroquine, intravenous immunoglobulin (IVIG), or mycophenolate mofetil added as adjuvant therapy if warranted (Table 1) [27].

### 2.3. Subacute Cutaneous Lupus Erythematosus (SCLE)

**Figure 3:**
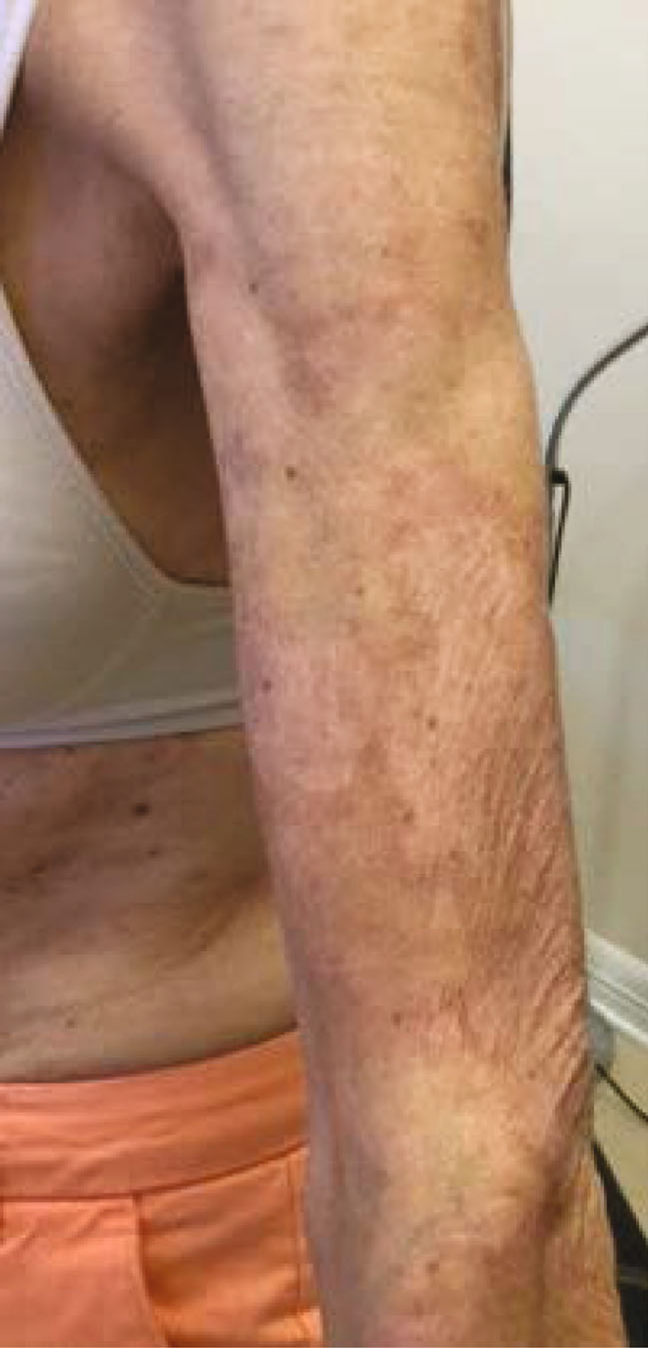
Subtle annular plaques on the left upper extremity of this female patient diagnosed with subacute cutaneous lupus erythematosus.

**Figure 4:**
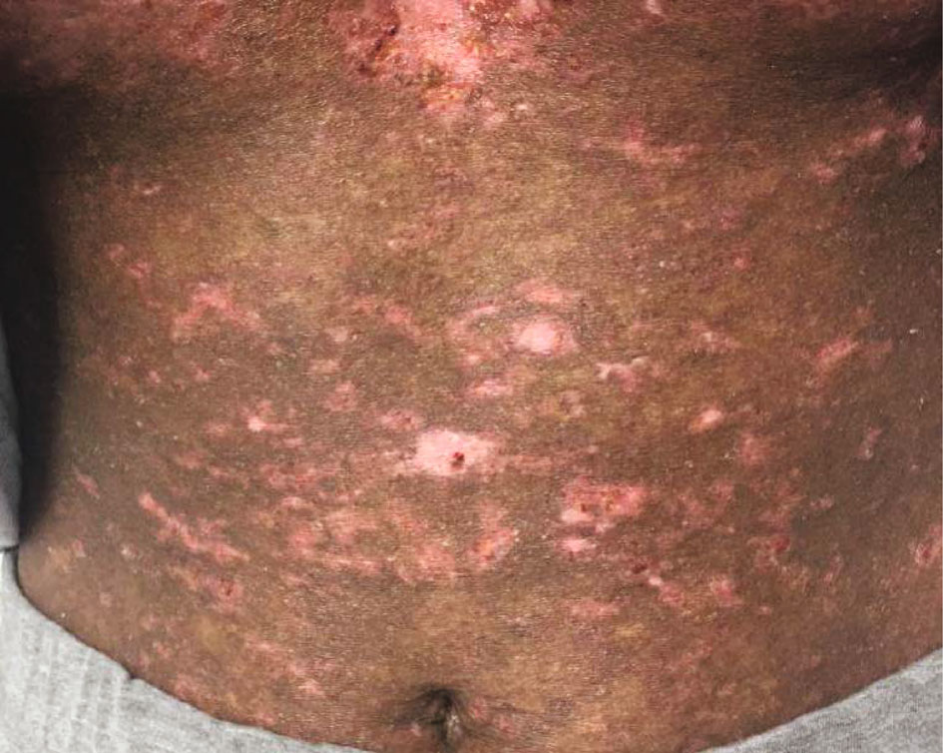
Dyspigmentation apparent during gradual resolution of this rash in subacute cutaneous lupus erythematosus.

This classification of CLE refers to a photosensitive eruption that has a longer duration than ACLE. This rash is typically distributed on sun-exposed skin, with a predilection for the upper torso, back, neck, and arms. The midface is usually spared [1]. Oral lesions, though rare, have also been reported [28]. Lesions may present as either erythematous or annular polycyclic lesions (Figure 3), or they may have a nonindurated, psoriasiform, papulosquamous appearance. The superficial nature of the inflammatory infiltrate seen in SCLE can often result in dyspigmentation (Figure 4), but scarring or dermal atrophy is rare [1, 29]. Of note, the Rowell syndrome refers to the presence of erythema multiforme-like lesions in lupus patients and can be categorized as a rare subtype of SCLE [30].

About 10-30% of cases of SCLE are drug-induced, with antihypertensives (e.g., hydrochlorothiazide, angiotensin-converting enzyme inhibitors, and calcium channel blockers), antifungals (e.g., terbinafine), tumor necrosis factor (TNF) inhibitors, antiepileptics, and proton pump inhibitors being the most common offending agents [1, 31, 32]. Patients with drug-induced SCLE often have an older age of onset of disease than those with idiopathic SCLE, which is likely secondary to increasing medication use with increasing age. Duration of drug use prior to the onset of cutaneous findings is most commonly weeks to months but may be as long as three years [31].

SCLE is highly associated with ANA and anti-SS-A/Ro positivity [33–35]. One recent retrospective review of 90 SCLE patients demonstrated 89% ANA positivity and 99% anti-SS-A/Ro positivity [33], while another Italian study demonstrated strong associations of SCLE with anti-SS-A/Ro, anti-Smith, and anti-ribonucleoprotein (anti-RNP) antibodies [36]. Caucasian race and smoking are also associated with increased risk of SCLE. As many as 20 to 50% of patients with SCLE will go on to meet criteria for SLE, but these patients tend to have a milder disease phenotype with less internal organ manifestations of disease than the typical SLE patient [37, 38]. SCLE may also be seen in patients with primary Sjӧgren's syndrome [33].

Histopathology of SCLE is similar to the typical findings shared by many CLE lesions: interface dermatitis and hyperkeratosis, basement membrane thickening, follicular plugging, and superficial and deep lymphocytic cell infiltrate [29]. However, when compared to other forms of CLE, epidermal changes and superficial lymphocytic infiltrate are much more common in SCLE. SCLE lesions also tend to have less hyperkeratosis, less basement membrane thickening, less periadnexal infiltrate, and less follicular plugging when compared to discoid lesions [39, 40]. Some studies have shown that deposits of IgG and IgM are found more frequently within the epidermis as opposed to the dermal-epidermal junction [39]. It is postulated that this is due to anti-SS-A/Ro autoantibody deposition within the epidermis [41]. When comparing the histopathology of drug-induced SCLE versus idiopathic SCLE, leukocytoclastic vasculitis is more prominent in drug-induced cases, and increased mucin deposition is more typical of idiopathic cases [32].

Treatment of SCLE lesions includes the use of topical steroids and/or calcineurin inhibitors. Oral antimalarial drugs are first-line systemic therapy for SCLE [6, 7].

### 2.4. Classic Discoid Lupus

Discoid lupus erythematosus (DLE) is one of the most common cutaneous manifestations of lupus and is categorized as a chronic cutaneous LE. Lesions are classically distributed on the face, scalp, and ears but may be more widespread in less than 20% of DLE cases [42]. Patients have increased risk for progression to SLE if widespread involvement is observed [43–45]. It is uncommon for a DLE patient to have lesions below the neck without concurrent head and/or neck findings. DLE lesions can also affect the lips, nasal mucosa, conjunctivae, and genitals [46]. Sun exposure seems to have a role in the development of lesions, but discoid lesions can be found on sun-protected skin [47]. Other triggers of DLE include trauma (Koebner effect), cold exposure, infection, dermatitis, ultraviolet light (UV) exposure, and thermal burns [6].

**Figure 5:**
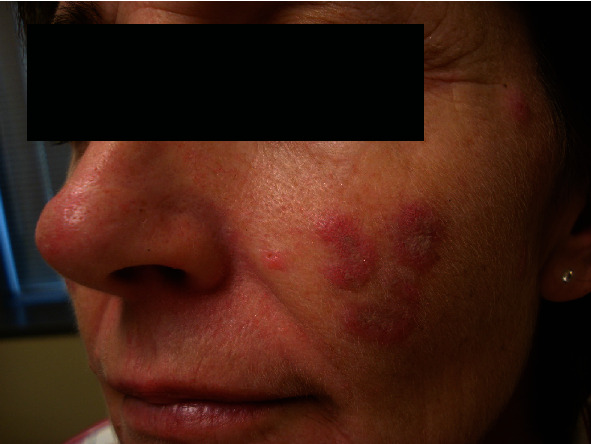
Coin-shaped erythematous plaques seen on this female patient's left cheek, biopsy results consistent with discoid lupus.

**Figure 6:**
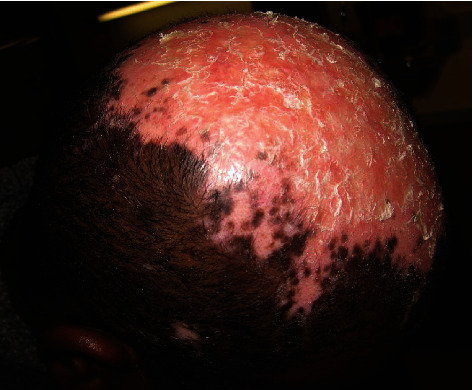
Scarring alopecia as a result of discoid lupus on the scalp of this male patient.

**Figure 7:**
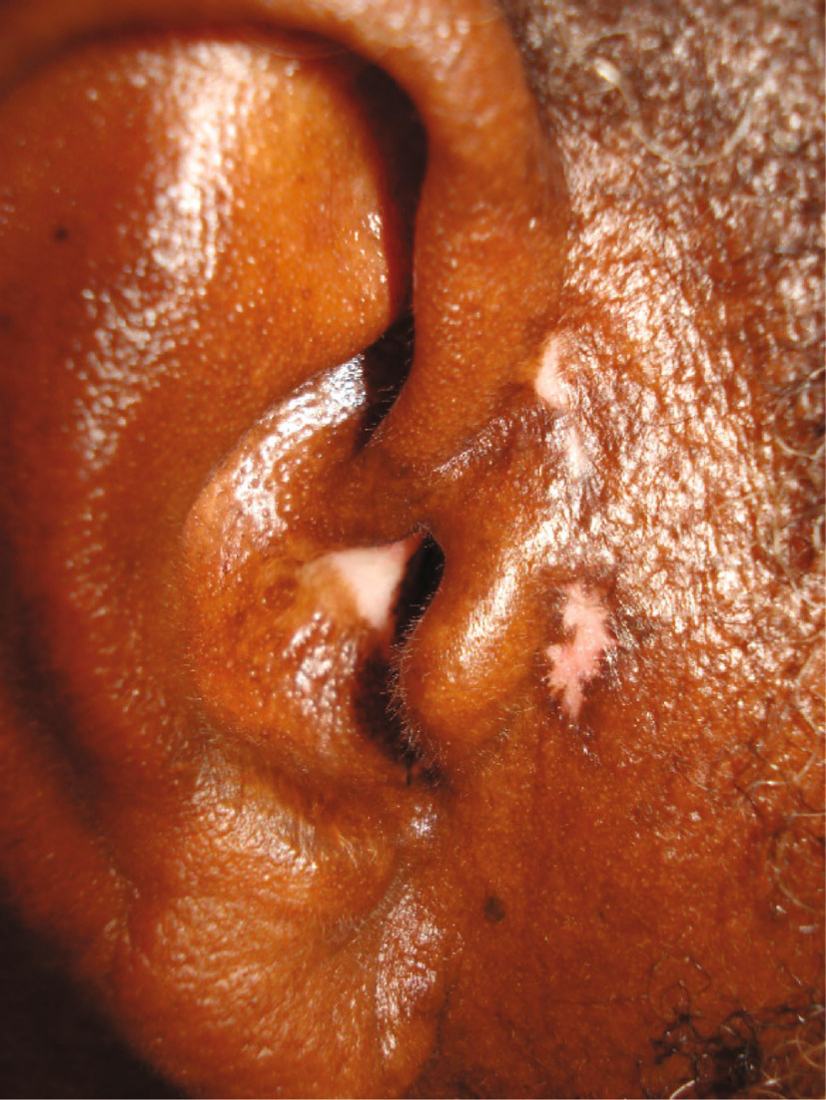
Hypopigmented small plaques with border of hyperpigmentation on the right preauricular region.

DLE cutaneous findings are characterized by variably sized coin-shaped erythematous plaques with adherent follicular hyperkeratosis and plugging (Figure 5). These lesions have a high potential for disfiguration or scarring [42]. The early indurated erythematous plaques of DLE can initially be mistaken for psoriasis, lymphocytoma cutis, cutaneous T cell lymphoma, granuloma faciale, polymorphous light eruption, and sarcoidosis, among many other dermatologic diagnoses [7]. Active lesions are inflammatory with superficial and deep dermal infiltrate, causing the lesions to feel thick and firm. Scalp plaques can result in scarring alopecia depending on severity and duration (Figure 6) [42]. Longstanding lesions often demonstrate various pigmentary changes, classically with hypopigmentation centrally and hyperpigmentation peripherally (Figure 7) [48]. Cases of squamous cell carcinoma developing within longstanding DLE lesions have been documented [49–51].

A recent review article demonstrated that up to 28% of DLE patients are at risk of developing SLE [43]. Reported factors that increased the likelihood of SLE progression include widespread DLE lesions, joint involvement, nail changes, anemia, leukopenia, high erythrocyte sedimentation rate, and elevated ANA titer [43]. Another study indicated that in addition to these factors, thrombocytopenia and the false-positive Wassermann reaction could be strong indicators of progression to disseminated DLE or SLE [45].

Histologic findings in DLE have some overlap with other CLE lesions, but overall, distinguishing features of active lesions include hyperkeratosis, vacuolar degeneration of the basal keratinocytes, significant follicular plugging, lymphocytic adnexal and deep perivascular infiltrates with subepidermal band, papillary dermal edema, initial atrophy, melanophages in the papillary dermis, and deposition of mucin among collagen fibers [7, 29, 40]. Pilosebaceous atrophy and more significant basement membrane zone thickening are more likely in DLE rather than in SCLE lesions [1]. Dermal fibrosis, adnexal atrophy, vessel dilatation, and presence of melanophages corresponding to scarring aspects are seen in more chronic, less active lesions [29]. In DLE patients, the dermal-epidermal junction demonstrates particulate staining on DIF rather than the epidermal staining seen in SCLE [40]. In fact, 90% of DLE lesions have a positive lupus band test, with C3 and IgM being the most common deposits [52]. DLE patients are less likely than other CLE patients to have positive ANA, double-stranded deoxyribonucleic acid (dsDNA), Smith, RNP, and anti-SS-A/Ro antibodies [52, 53].

Topical steroids are considered first-line therapy for DLE, and the presence of discoid lesions is one of the few instances in which it is recommended to use high-potency topical steroids on the face (Table 1) [54]. One recent literature review found that fluocinonide cream may be more effective than hydrocortisone in clearing discoid lesions, though patients were advised to limit treatment to two to three consecutive weeks and to be aware of side effects (discussed below) [55]. Topical calcineurin inhibitors are useful in thin-skinned areas where corticosteroids are inappropriate [54]. In active, refractory discoid lesions, monthly intralesional triamcinolone can be effective [56]. Systemic therapy is often warranted in DLE cases unresponsive to topical and intralesional therapy, or in patients with extensive disease. In these cases, antimalarials are the mainstay of treatment. Second-line systemics include methotrexate, systemic retinoids, thalidomide, lenalidomide, dapsone, adjuvant mycophenolate mofetil, azathioprine, and intravenous immunoglobulin (IVIG) [54].

Preventative measures should also be taken in DLE patients. Sun protection is an important component of therapy for chronic discoid lesions or in hypopigmented skin, where the risk of skin cancer development is higher [7]. Smokers may have more extensive cutaneous disease and may be more difficult to treat as antimalarials appear to be less effective in these patients, making smoking cessation a vital component of treatment [7, 54, 57].

### 2.5. Hypertrophic, Verrucous Discoid Lupus

Hypertrophic DLE is an unusual variant of DLE, representing only two percent of the lesions seen in CLE [52]. It is often clinically described as papulonodular or hyperkeratotic [58], and its characteristic bright red appearance can mimic a cutaneous neoplasm [29]. These lesions are frequently seen on the extensor surfaces of the upper extremities, but involvement of the face and upper trunk has been reported [59]. These lesions can present concurrently with typical discoid lesions on other sites of the body, which often aids in diagnosis [1, 52].

Histologically, these lesions typically demonstrate one of two patterns. The first demonstrates acanthosis, hyperkeratosis, and papillomatosis with a band-like mononuclear cell infiltrate in the upper dermis resembling hypertrophic lichen planus. Within this first subset of lesions, the granular layer is thickened, and many eosinophilic dyskeratotic cells can be found in the lower epidermis [59, 60]. The second pattern demonstrates focal epidermal acanthosis with deep dermal projections with only a sparse lichenoid cellular infiltrate and shares characteristics with a keratoacanthoma [58–60]. Both patterns can present in conjunction with diagnostic features of LE such as basement membrane thickening, hydropic degeneration of the basal cell layer, and perivascular and periadnexal lymphocytic infiltrate.

In addition to treatment options described above for classic DLE, both topical and systemic retinoids can be effective in treating hypertrophic DLE (Table 1) [52].

### 2.6. Mucosal Discoid Lupus

Oral involvement in CLE cases ranges from 3 to 25%. Typical clinical presentation is a plaque or erosion with central white papules and white radiating striae [61]. The characteristic histopathology seen in mucosal lupus lesions include interface mucositis with lymphocytic infiltrate, necrotic keratinocytes, and hydropic degeneration of the basal layer. DIF is frequently positive, often demonstrating linear deposition of IgM, IgG, and C3 in the basement membrane zone [62].

There is a risk of development of squamous cell carcinoma (SCC) within mucosal lupus lesions, most frequently in lip lesions [63–65], but cases of SCC developing on the palate, gingiva, and other mucosal surfaces also exist within the literature [66]. Mucosal discoid lupus lesions should be monitored closely for development of malignancy.

### 2.7. Lupus Erythematosus Tumidus (Tumid Lupus)

Tumid lupus is a form of chronic CLE that recurs and remits in response to sun exposure and has a mild male predilection when compared to other forms of CLE [7, 67, 68]. In this presentation of lupus, erythematous polycyclic plaques with raised borders and smooth surfaces are typically the presenting feature. These plaques lack scale or follicular plugging and can have central clearing, and the epidermis appears to be uninvolved in pathology. The cutaneous findings are sometimes described as “urticarial plaques” but these fixed plaques should not be confused with urticarial vasculitis [1]. The plaques typically occur on the face or trunk, or on sun-exposed areas [29, 67, 68]. Clinically, lesions are similar to lymphocytic infiltrate of Jessner, a benign lymphocytic infiltrate of the skin presenting as asymptomatic erythematous papules or annular plaques on the head and upper trunk in middle-aged adults [1]. Some believe that tumid lupus and the lymphocytic infiltrate of Jessner are the same disease or at least very closely related [69].

There is a low prevalence of SLE in LE tumidus patients, and the lack of immunoglobulin deposition within lesions has caused many to debate whether LE tumidus is truly a form of cutaneous LE or if this is an independent disease process. LE tumidus lesions often resolve without residual scarring or chronic skin changes [1, 67, 68], but reports of tumid lupus patients later developing lesions characteristic of other types of CLE have been reported [67, 68, 70, 71].

Histopathology of the lesions demonstrates intense dermal perivascular and periadnexal inflammatory infiltrates [67, 68]. In LE tumidus, DIF is often negative or nonspecific [1, 29], but in more chronic lesions, IgG and IgM may be found along the basement membrane. Treatment options include topical corticosteroids (first line) and systemic antimalarial therapy (Table 1) [67, 68]. Some cases of spontaneous resolution of lesions have also been reported [67].

### 2.8. Lupus Panniculitis (Lupus Profundus)

Lupus panniculitis only makes up about 2-3% of cases of CLE [72], usually occurring in adults with median onset between ages 30 and 40 [73], although an association with neonatal lupus has been described [74]. There is a mild female predilection in this condition.

**Figure 8:**
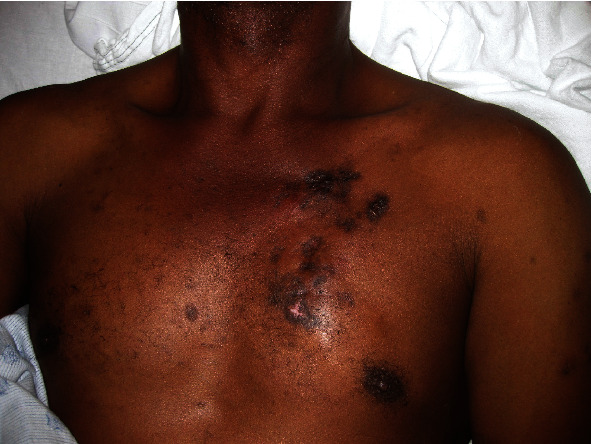
Multiple tender, indurated hyperpigmented plaques affecting the left chest in this patient with lupus panniculitis.

Lupus panniculitis is a result of inflammation of fat and presents as tender, indurated plaques that can disfigure patients (Figure 8). These lesions are typically distributed over the face, scalp, upper arms, upper trunk, buttocks, and upper thighs, while the distal extremities are notably spared [73]. Discoid lesions may present on the skin overlying the panniculitis in as many as half to two-thirds of cases but are sometimes too subtle to be recognized clinically and are instead noted on histopathological examination. The overlying skin can also feel bound down to the subcutaneous nodule or plaque, creating depression in the skin and often leading to ulceration and finally subcutaneous atrophy [1].

Histopathology of lupus panniculitis demonstrates nodular aggregates of lymphocytes, hyaline necrosis of fat lobules, lymphocytic vasculitis, and mucin or calcium deposition. Granulomas are sometimes present along the septa, but this is typically not a prominent feature. DIF identifies a positive lupus band in most cases [74].

Lupus panniculitis tends to have a chronic course with many relapses and remissions. Lesions can be debilitating but typically do not affect long-term survival of patients. Lupus panniculitis may either precede or follow other forms of chronic cutaneous LE and is unlikely to progress to systemic LE. If, however, progression to SLE does occur, patients typically have mild systemic manifestations, such as arthralgias or the Raynaud phenomenon [72]. Patients can present with lower-titer ANA as well as other extractable nuclear antigens [72].

Antimalarials have been shown to have some efficacy in lupus panniculitis, and given its typically chronic course, treatment may be required for several years. Systemic corticosteroids are reserved only for treatment of the initial phases of lupus panniculitis. Other systemic therapies include dapsone, mycophenolate mofetil, cyclophosphamide, thalidomide, and IVIG [75]. Rituximab has emerged in several case reports as a potential option for treatment as well [76–78]. Overlying discoid lesions can be treated with topical or intralesional steroids as discussed above.

### 2.9. Chilblain Lupus Erythematosus

**Figure 9:**
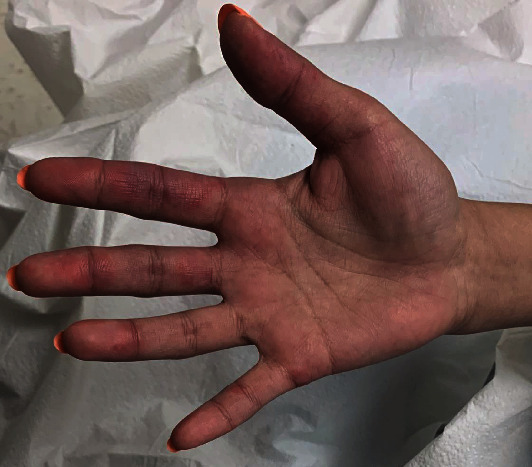
Painful, subtly dusky papules and plaques on the fingers of a patient with chilblains lupus.

Chilblain lupus (CHLE) is a rare form of chronic CLE that clinically resembles frostbite. It is triggered by cold temperatures and presents with painful violaceous or dusky papules, plaques, and nodules in cold-exposed areas such as the fingers (Figure 9), toes, heels, and more uncommonly the nose and ears [6, 7, 79]. These lesions can develop central erosions or ulcerations [7].

There is an autosomal dominant familial form of chilblain lupus with onset during childhood that is a result of heterozygous mutations in *TREX1* or *SAMHD1* [1, 79, 80] which upregulate type I interferon signaling [81]. These patients may have positive ANA or arthralgia but do not usually progress to SLE. Sporadic CHLE typically presents in middle-aged females rather than children and may be accompanied by discoid lupus [79, 82, 83], Raynaud's phenomenon, and livedo reticularis [79, 84]. In spontaneous CHLE, progression to SLE has been documented in as high as 18% of cases [79]. Antibodies to SS-A/Ro can be found in a subset of sporadic CHLE patients [84].

Histopathology is remarkable for epidermal atrophy, interface vacuolization, and perivascular mononuclear infiltrate [7]. Other features of cutaneous lupus including deposition of IgM, IgA, and C3 with perivascular deposits of C3 and fibrinogen are also found on DIF of chilblain lupus lesions [79].

Many patients respond well to protection from the cold and treatment of infected necrotic areas with antibiotics. Topical steroids are next-line therapy, followed by systemic steroids, and finally calcium channel blockers which counteract the possible pathogenic influence of vasoconstriction in CHLE [79]. Many cases of CHLE are unresponsive to antimalarials [23]. Successful treatment with mycophenolate mofetil has been reported in refractory spontaneous cases [83, 84]. Baricitinib alleviated symptoms in 3 patients with familial CHLE [81, 85], and use of ruxolitinib has successfully treated two cases of CHLE [86, 87].

### 2.10. Lichenoid Cutaneous Lupus Erythematosus-Lichen Planus Overlap Syndrome

Cutaneous LE and lichen planus (LP) are distinct dermatoses that can in some circumstances occur as an overlap syndrome, or as a syndrome with mixed clinical and histopathological features of both LE and LP [88]. There is some controversy regarding the definition of this rare syndrome. Some experts suggest that true LE/LP overlap is defined as the presence of LE and LP within the same lesion [88, 89]. However, many believe the coexistence of LE and LP lesions in the same patient could be recognized as an overlap syndrome. The diagnosis of the condition is based on combined clinical, histological, and/or immunopathological features of both diseases simultaneously.

This is a rare condition, but most cases occur between ages 25 and 45 with a slight female predominance [90]. Of the reported cases of LE/LP overlap syndrome, there seem to be two different clinical presentations. The first presentation includes painful, blue-red, scaly, centrally atrophic plaques on the extremities, while the second presents as verrucous, papulonodular lesions on the upper extremities and hands [89, 91]. Lesions located in different sites have also been reported in the literature, including mucous membrane, scalp, and nail involvement, but these cases are not as common. The course of disease is often chronic [91]. Isoniazid, procainamide, and acebutolol have each been reported as inciting agents in isolated cases of LE/LP overlap syndrome [91–94].

Histopathology of the overlap syndrome may demonstrate features of either LP or LE or features of both simultaneously [89]. Both LE and LP demonstrate histopathological findings of colloid bodies and basement membrane changes. Colloid bodies are deeper and more abundant in LP, and basement membrane cleft formation is also more commonly seen in LP. On the other hand, thickened basement membrane zones are more frequently seen in LE [90, 91].

Histopathology is sometimes insufficient for diagnosis of the overlap syndrome due to the significant overlap between LE and LP, so DIF can be helpful. DIF of lichen planus demonstrates cytoid bodies staining for IgM (or sometimes IgG) along with fibrin and fibrinogen in a band-like fashion along the basement membrane zone [90, 91]. DIF of discoid lupus lesions also demonstrates IgM staining of cytoid bodies but usually displays immunoglobulin and complement deposition at the dermal-epidermal junction [90]. In the overlap syndrome, DIF may demonstrate immunoglobulin deposits in cytoid bodies or linear fibrinogen at the basement membrane zone along with other distinct features of either LP or LE [91].

Topical tacrolimus, systemic retinoids, and cyclosporine have been shown to be efficacious in LE/LP overlap syndrome [88, 90, 91]. Follow-up for systemic disease in these patients is necessary as there is a reported conversion to SLE in about 5-10% of cases [95].

### 2.11. Bullous Lupus

**Figure 10:**
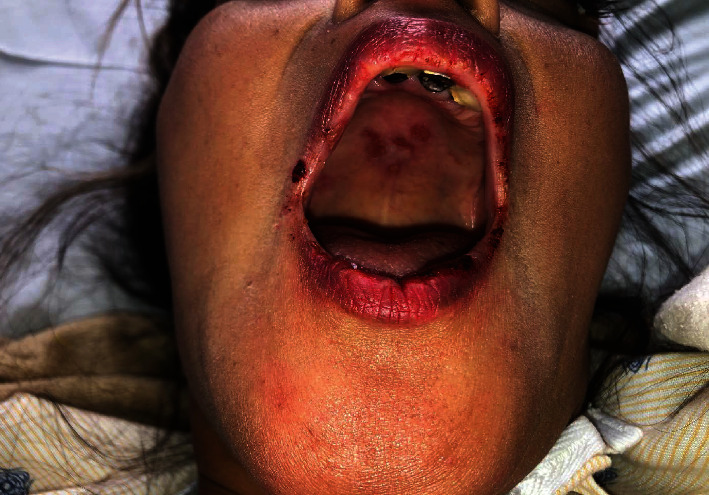
Involvement of the lips and palate in a case of bullous lupus.

**Figure 11:**
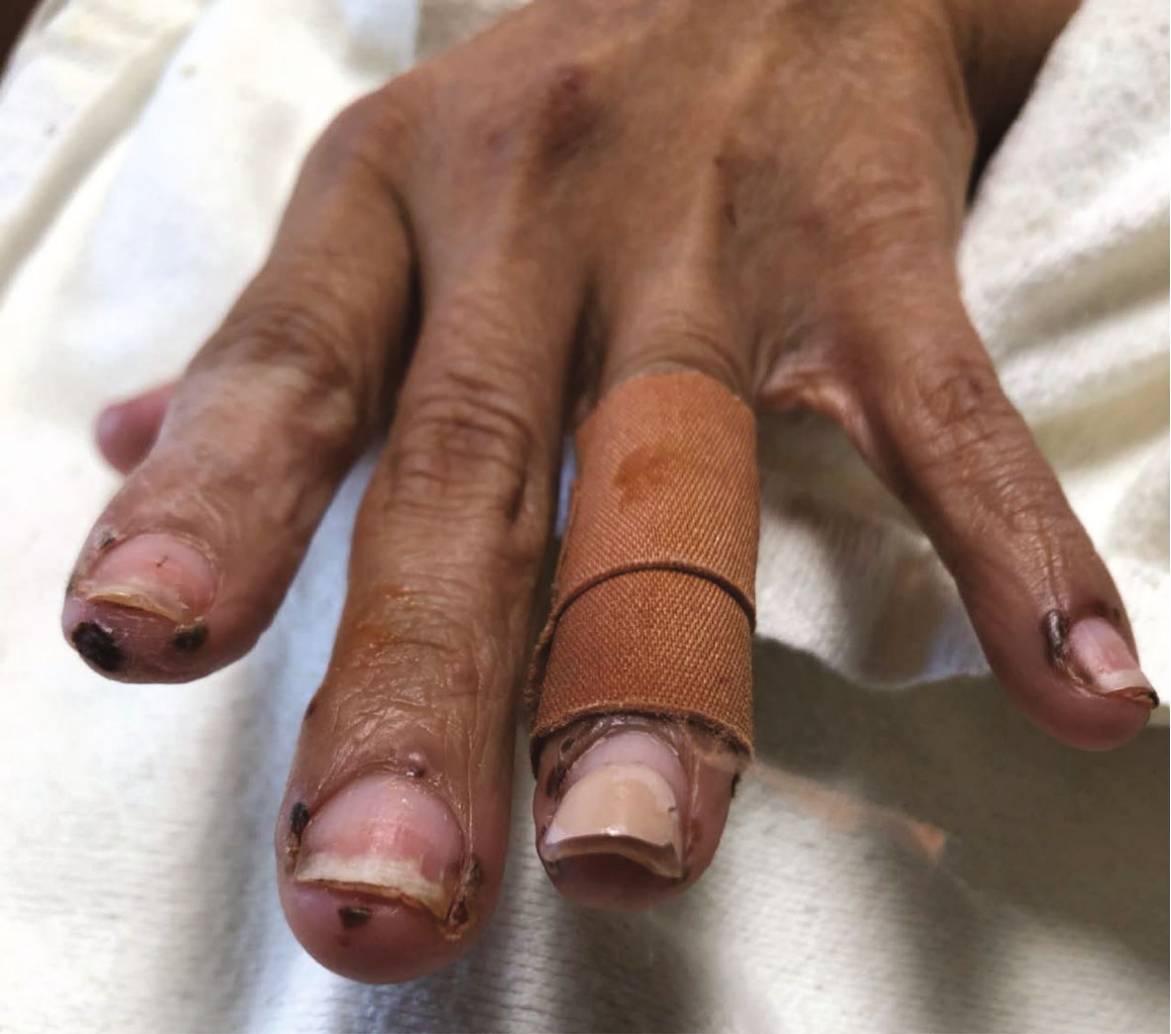
Small hemorrhagic vesicles on the hands of a bullous lupus patient.

Bullous lupus is found in less than five percent of patients with SLE. Bullae may be found on any part of the body, but there is a predilection for sun-exposed areas (face, chest, upper extremities, vermillion border, or oral mucosa, as seen in Figure 10) [96]. Bullae or vesicles can be found on both erythematous and nonerythematous bases (Figure 11), typically heal without scarring, and are not particularly pruritic. Most patients with bullous lupus develop antibodies to type VII collagen, which is a shared antigen in epidermolysis bullosa acquisita. Other clinical, histologic, and immunologic features of bullous lupus help distinguish between these two cutaneous disorders. The differential diagnosis for bullous lupus also includes dermatitis herpetiformis, bullous pemphigoid, and linear IgA bullous dermatosis. Other noncutaneous clinical features of SLE and DIF IgG subtyping allow for distinction among these entities [96, 97].

Of note, it is important to differentiate other cutaneous manifestations of lupus that create epidermal detachment (such as the TEN variant of LE described above) from bullous lupus, primarily for treatment purposes, as bullous lupus often has dramatic response to dapsone while other variants do not. While antibodies to type VII collagen may develop in bullous lupus, the TEN variant of lupus results from excessive interface dermatitis within severe CLE lesions. Subsequent severe hydropic degeneration of the basal layer of the epidermis can lead to bullae formation, and if this event is exaggerated with massive apoptotic injury, the TEN-like acute CLE is the rare result [96].

Skin biopsy in bullous lupus reveals a predominance of neutrophils in the upper dermis with microabscesses within the dermal papillae, subepidermal blistering, and a perivascular inflammatory infiltrate and mucin deposition in the dermis. Mucin, as with many variants of cutaneous lupus, is a distinguishing feature of the histopathology. DIF is positive, with mainly IgG and/or IgM with C3 at the dermoepidermal junction [96, 97].

Bullous lupus responds dramatically to low-dose dapsone. In cases of nonresponse, steroids, antimalarials, and other immunosuppressants can be efficacious [96, 98]. Rituximab may be effective for refractory cases [96, 98, 99].

### 2.12. Neonatal Lupus

Mothers who have anti-SS-A/Ro antibodies have about a 2% risk of having a child with neonatal lupus (NLE), with a recurrence rate of about 20% with each subsequent child [100]. Some mothers of newborns with NLE may have primary Sjӧgren's syndrome or SLE, but in many cases, the mother is paucisymptomatic or asymptomatic [100]. Almost all babies with NLE have anti-SS-A/Ro antibodies, and there are some reports of anti-RNP positivity in these patients [101].

Earlier data showed an increased prevalence of NLE in female infants, but more recent studies demonstrate roughly equal incidence in boys and girls [100, 102]. Less than five percent of patients with neonatal lupus develop SLE later in life [100].

NLE can be categorized as a neonatal form of subacute LE, but unlike SCLE in adults, skin lesions of NLE often occur on the face, especially periorbital and scalp regions. This distribution exhibits the role of photosensitivity in rash development, though it is possible for lesions to be present at birth [1, 100]. The rash is typically macular annular or demonstrates elliptic erythema, with papules or plaques occasionally observed [100]. Lesions often resolve without scarring, although residual dyspigmentation or telangiectasias can persist [1].

NLE skin lesions occur in about 40% of cases, and other clinical features include liver dysfunction, cytopenias (especially thrombocytopenia), and cardiac arrhythmias. Hepatobiliary disease can vary from liver failure during gestation or in the newborn, conjugated hyperbilirubinemia in the few weeks following birth, or elevated aminotransferases at two to three months of age [1]. Cardiac arrhythmias are seen in only about 25% of cases [100], but the mortality rate of cardiac NLE is about 20%, and the majority of affected newborns require pacemakers. There are also some reports of hydrocephalus, microangiopathic hemolysis, and disseminated intravascular coagulation in infants with internal organ manifestations of NLE [1]. While extracutaneous involvement is uncommon in NLE, evaluation for such is necessary in all infants with NLE given the risks of 4untreated internal organ manifestations of disease.

Management of infants without cardiac arrhythmias (e.g., complete heart block) includes avoidance of sun exposure and laser therapy for residual telangiectasias [1, 100]. Topical steroids or antimalarials are typically not advised, as most manifestations of disease spontaneously resolve. However, for atrioventricular heart block, counseling and fetal and maternal screening are all necessary as there are limited options for atrioventricular block *in utero*. Management in these cases is primarily expectant, and more than 90% of these infants eventually require pacemaker placement [100].

## 3. Treatment

### 3.1. Photoprotection

Patient education regarding heat, sun, and certain drug exposures is important for all types of CLE [7]. Sunscreen adherence is a very important component of therapy, as both UVA and UVB radiation have been shown to induce CLE lesions [103, 104]. At least 2 mg/cm^2^, or about a quarter of a teaspoon per the average face, of a sunscreen with sun protection factor (SPF) of at least 50 should be applied 20-30 minutes to skin before sun exposure [104]. Physical sunscreens like zinc oxide or titanium dioxide provide broad-spectrum protection against UVA and UVB rays. Many other commercial sunscreens protect primarily against UVB only, so choosing one that explicitly advertises broad-spectrum coverage is important. Of note, UVA rays, which penetrate glass, can reach patients through windows while indoors or while driving, and patients should be counseled accordingly [105].

Further steps include counseling patients on avoidance of sunbathing or travel to places near the equator [2]. Indoor fluorescent lighting provides some increased risk of exacerbating CLE, and patients should also be encouraged to shield bulbs [106, 107]. With avoidance of UV rays however, 25-vitamin D levels should be monitored, and some experts advise supplementation with at least 400 IU of vitamin D3 daily [108].

It should also be noted that while cutaneous lupus lesions themselves can progress or worsen with sun exposure, certain systemic agents used to treat CLE can cause photosensitivity, potentially enhancing UV ray induction of CLE lesions. These systemic agents mentioned include hydroxychloroquine, methotrexate, azathioprine, leflunomide, and nonsteroidal anti-inflammatory drugs, among others. Patients should be counseled on this enhanced risk of sun sensitivity when started on these agents.

### 3.2. Smoking Cessation

Smoking cessation is paramount in CLE management. Smoking is a risk factor for development of many other autoimmune diseases, such as rheumatoid arthritis, primary biliary cirrhosis, and Graves' disease [109]. Cigarette smoke's toxic agents may cause genetic mutations and negatively influence the body's immune responses [110]. Many studies have demonstrated that smoking is more prevalent among CLE patients, as well as associated with more severe disease in CLE patients. Furthermore, the CLE skin lesions of patients who smoke are more likely to be refractory to treatment with antimalarials [111–113]. Historically, epidemiologic studies of smoking and SLE risk have been somewhat conflicting [109], with elevated SLE risk among current smokers compared to nonsmokers, but not among past smokers compared to nonsmokers [114, 115]. More recent studies have confirmed that both current and past smokers do indeed have an elevated risk of SLE [109, 110]. Enough evidence exists in the literature to warrant smoking cessation as a cornerstone of management in CLE.

### 3.3. Topical Therapy

Topical steroids may be helpful in treating CLE but are usually insufficient as monotherapy. Medium-potency triamcinolone 0.1% to high-potency clobetasol propionate 0.05% may be used twice daily for two weeks followed by a one- to two-week rest period if attempting to limit side effects [6]. High-potency steroids for discoid lesions on the face may be necessary, though the risk of skin atrophy must be taken into account [1, 6]. Ointments, creams, foams, lotions, solutions, and gels are all options, and the choice of vehicle can be based on potency and patient preference. Ointments in general provide more occlusion and are therefore more easily absorbed compared to creams. While ointments are helpful for affected dry, hyperkeratotic areas like the hands and feet, ointments can cause cutaneous side effects on the thin skin on the face or within skin folds like the axillae or groin. Solutions, foams, and gels are typically better tolerated on hairy areas of skin than are ointments or creams. Patients must be advised about the risks and benefits of topical steroids and counseled to monitor for cutaneous side effects such as skin atrophy, striae, telangiectasias, easy bruising, and hypertrichosis [1].

Monthly intralesional triamcinolone can be effective in treating active discoid and LE tumidus lesions [1]. Triamcinolone is typically injected in concentrations of 5 to 10 mg/mL, and the dose is dependent on the thickness and surface area of the lesion being treated [6]. Topical tacrolimus ointment and pimecrolimus cream have both shown some efficacy in treating CLE and are useful for atrophy-prone areas such as the face, eyelid, intertriginous, and groin regions, though thicker lesions of DLE are less likely to respond [6]. Topical retinoids like tretinoin and tazarotene can be useful for hyperkeratotic lesions sometimes seen in DLE, though skin irritation may occur with these agents [6].

### 3.4. Antimalarials

Oral aminoquinolone antimalarial agents are efficacious and relatively safe for use in CLE, and they have remained the gold standard for systemic therapy for at least half a century. Hydroxychloroquine sulfate is most frequently chosen, with chloroquine and quinacrine (mepacrine) as second options [1]. Routine ophthalmologic monitoring is required while taking these agents, with cumulative dose increasing chances of ocular toxicity over time [116]. Concomitant use of hydroxychloroquine and chloroquine is not recommended due to increased cumulative risk of retinal toxicity [7]. Quinacrine does not cause ophthalmologic toxicity, but there is a risk of aplastic anemia with higher doses of this medication [7]. Quinacrine is not commercially available in the United States or Canada but may be compounded by certified compounding pharmacies. Antimalarials are contraindicated in patients with hypersensitivity to 4-aminoquinolones, preexisting retinopathy, and myasthenia gravis [117]. The most common side effects of antimalarial agents are gastrointestinal upset, xerosis, and skin hyperpigmentation, but ocular toxicity is the most emphasized complication in the literature [118].

It should be noted that CLE response to antimalarials is gradual, and patients need to be counseled in kind. Clinically, it takes two to three months for visible change to be appreciated, with even more time required to achieve maximal efficacy [1]. While antimalarials have good bioavailability after ingestion, especially if taken with a fatty or protein-rich meal, these agents accumulate in melanin-containing retina and skin. This results in long drug half-lives (40-50 days) [117], with slow pharmacokinetics delaying onset of action. When initiating antimalarial therapy for cutaneous LE, topical or intralesional agents can be administered concurrently to provide more immediate results.

A recent meta-analysis found a pooled response rate to antimalarials of 63% in CLE cases [103]. Chasset et al. found that the cutaneous response rate to antimalarials was higher for ACLE than for SCLE and lupus tumidus, with a low rate of response in chilblain lupus [23]. However, results may have been skewed by an increased use of concurrent systemic steroids in ACLE patients.

### 3.5. Methotrexate

Methotrexate may be used as second-line agent in patients with refractory CLE [6, 117, 119]. Studies have demonstrated positive clinical response in patients treated with low-dose methotrexate after hydroxychloroquine treatment failure, and in general, methotrexate has an excellent safety profile [120, 121]. Methotrexate is teratogenic, and the importance of contraception while taking this drug cannot be stressed enough. Gastrointestinal upset, fatigue, hair loss, and oral ulcers are commonly reported adverse effects but may be mitigated by subcutaneous administration, or higher doses of prophylactic folic acid, respectively. Lalani et al. recently demonstrated with a meta-analysis relatively low prevalence of stomatitis and alopecia (between 5.7 and 8.0% and between 1.0 and 4.9%, respectively) in patients taking methotrexate [122]. Bone marrow suppression and hepatotoxicity may occur, and routine lab monitoring is required while on therapy to ensure patient safety [6].

### 3.6. Azathioprine

Azathioprine is another second-line agent used in refractory CLE, though less efficacious than methotrexate [117]. However, azathioprine is safe for use during pregnancy, making it an attractive option in certain cases [119, 123]. Not many studies exist to demonstrate the efficacy of azathioprine in cutaneous lupus, but several case series support its use after other agents have failed [123, 124]. Routine lab monitoring is required while on therapy to monitor for bone marrow suppression and hepatotoxicity. Though rare, acute hepatitis and agranulocytosis may occur [124].

### 3.7. Mycophenolate Mofetil

Mycophenolate mofetil (MMF), an inhibitor of T and B cell proliferation and autoantibody production, has also been used in refractory CLE with some success. A recent study on MMF in SLE patients, 57 of whom held diagnoses of both CLE and SLE, demonstrated resolution of either rash, alopecia, or mucosal ulcers in 27 of the affected patients within 12 months, though patients were also receiving concomitant corticosteroids [125]. A Scandinavian systematic review found a favorable response in a majority of MMF-treated CLE patients (68.8%) [126]. Another encouraging retrospective study from the *Journal of the American Academy of Dermatology* supported the use of adjuvant MMF in refractory CLE [127]. However, a recent controlled trial did not show statistically significant improvement in CLE with MMF, though the study had a small sample size and was likely not adequately powered to demonstrate a response [128]. Differing study results may be attributable to the heterogeneity of cutaneous lupus manifestations, simultaneous use of other therapies, and differences in dose and duration of MMF treatment.

### 3.8. Belimumab

Belimumab is a monoclonal antibody that inhibits B-lymphocyte stimulator (BLyS), thereby inhibiting B cell activation [129]. Belimumab is one of the few Food and Drug Administration- (FDA-) approved drugs for lupus. Recent studies revealing utility of belimumab as an adjuvant treatment for skin manifestations of lupus have been highly promising [129–135]. A recent publication demonstrated that patients with isolated CLE all had significant improvement in their cutaneous disease, but overall, SLE patients with skin involvement as a group did not demonstrate statistically significant improvement [130]. Clinical response in this study was better in patients with mild persistently active lesions and for phototypes IV-VI [130]. One extensive literature review showed that certain patients may benefit from adjuvant belimumab more than others, including those with low serum complement and positive dsDNA antibodies [135]. This niche of patients has statistically significant improvement in mucocutaneous, musculoskeletal, immunologic, and hematologic manifestations of disease [135].

### 3.9. Other Agents

CLE that is refractory to antimalarials and agents listed above is particularly difficult to treat. Several other agents have been reported in the literature, with evidence typically limited to case reports. These include apremilast, ustekinumab, IVIG, rituximab, thalidomide, and dapsone [6, 7, 50, 134]. Rituximab may be less efficacious in chronic CLE [119]. Thalidomide can be effective in certain cutaneous disease but, due to its many side effects, should be reserved as a rescue therapy in refractory cases [119]. Dapsone has little success in CLE, except in cases of bullous lupus [97, 119]. Anifrolumab, a human monoclonal antibody to type I interferon receptor subunit 1, did reduce skin manifestation severity in the TULIP-2 trial [136, 137]; further studies are needed to determine how useful this drug will be for CLE.

## 4. Other “Lupus” Dermatologic Conditions

### 4.1. Lupus Vulgaris

Only one to two percent of all extrapulmonary tuberculosis cases demonstrate cutaneous involvement [138]. Lupus vulgaris, or tuberculosis luposa, is a rare form of cutaneous tuberculosis that makes up only 10-15% of cutaneous tuberculosis (TB) cases. Female predominance in lupus vulgaris is 2 to 3 : 1 [1]. Lesions are often a result of direct extension, or hematologic/lymphatic spread of TB, or autoinoculation with the Bacillus Calmette-Guerin (BCG) vaccine. The tuberculin skin test in these patients is usually positive [1, 135, 136]. Lupus vulgaris typically presents as patches and plaques, and these plaques are often psoriasiform in appearance [138]. Lupus vulgaris is most commonly seen on the face, where it can be clinically difficult to differentiate from discoid lupus [138]. Disseminated lupus vulgaris is very rare and presents as a granulomatous folliculitis [138].

Histology is remarkable for well-developed granulomas with scarce caseation and nonspecific inflammatory infiltrate [139]. Usually no acid-fast bacilli are visible on histopathology [139]. Diagnosis is made by a combination of histology, culture polymerase chain reaction (PCR), and interferon-gamma release assays (IGRAs) [1]. Treatment of the underlying TB infection is recommended [139].

### 4.2. Lupus Miliaris Disseminatus Faciei

Lupus miliaris disseminatus faciei (LMDF) is a rare, chronic, inflammatory dermatosis that mainly affects the faces of young adults of both sexes [140]. Some experts consider LMDF to be a severe form of granulomatous rosacea (GR) given the perifollicular localization of granulomas on histology. However, LMDF has some distinct features from GR, such as involvement of extrafacial sites and lack of erythema, telangiectasia, or ocular symptoms [141]. LMDF also results in chronic scarring, which is not true of GR. LMDF differs from GR on histopathology, given that large granulomas with necrosis are evident in LMDF lesions and the granulomas seen in GR are small and devoid of necrosis [141]. Given the caseous necrosis noted on histology, LMDF historically was thought to be a variant of lupus vulgaris. However, staining and PCR for *M. tuberculosis* is consistently negative in LMDF, and antitubercular drugs are not efficacious in LMDF [141]. The exact etiology of LMDF remains unknown, and its categorization as a granulomatous condition affecting the face is still debated [140].

LMDF presents with many yellow-brown to red papules and nodules, typically affecting the periocular or central facial regions, and demonstrates an “apple-jelly” appearance on diascopy [140, 142, 143]. Facial scarring in this disease process can often be permanent. Extrafacial involvement does occur in LMDF, which can cause clinical difficulty in distinguishing LMDF from sarcoidosis, cutaneous tuberculosis, or the necrobiotic form of granuloma annulare [1].

As mentioned previously, histopathology is remarkable for dermal granulomas with frequent central caseating necrosis [142]. Staining for organisms is always negative. Treatment strategies are broad, and reports of use of long-term topical steroids, minocycline, dapsone, oral steroids, intralesional steroids, isotretinoin, clofazimine, tranilast, cyclosporine, and laser all exist in the literature [140, 143]. LMDF most commonly has an indolent and self-limiting course with spontaneous resolution over one to four years despite residual scarring [140]. No treatment seems to be consistently efficient in preventing scarring caused by LMDF [140].

### 4.3. Lupus Pernio

Lupus pernio is a rare, late cutaneous presentation of sarcoidosis. Sarcoidosis is a multisystem disorder most commonly affecting young adults. Though involvement of nearly all parts of the body has been reported, the lymph nodes, lungs, eyes, skin, and liver are most commonly affected. Lupus pernio demonstrates a female prevalence and is more common in West Indian or African-American sarcoid patients than white patients [144]. Lupus pernio is associated with chronic sarcoidosis of the lungs in about 75% of patients and upper respiratory tract involvement in about 50% of patients [145].

Lupus pernio presents as chronic violaceous papulonodules to large plaques with scale that typically appear on the nose, cheeks, and ears [144]. These lesions can be complicated by nasal ulceration and septal perforation, which can be aggravated further by surgical intervention [144]. Lupus pernio rarely resolves spontaneously and can result in facial disfigurement as well as nasal obstruction or fibrotic pulmonary complications if the nasal cavity and maxillary sinuses become more extensively involved [146].

Histopathology of these lesions demonstrates changes that can be found in all organs affected by sarcoidosis: noncaseating granulomas with a sparse lymphocytic component referred to as “naked granulomas” [146]. These granulomas are typically found in the dermis but can be subcutaneous.

Treatment of lupus pernio can be challenging given the unpredictable course of disease. Therapeutic options include local, intralesional, and, if needed, systemic corticosteroids, as well as methotrexate, chloroquine, hydroxychloroquine, azathioprine, cyclophosphamide, thalidomide, infliximab, and even laser therapy [144, 146–150]. Lenalidomide has been reported to be successful in one refractory case of lupus pernio [151].

## 5. Conclusions

Cutaneous lupus erythematosus is an umbrella term for a diverse array of rashes with distinct clinical phenotypes, histopathology, and treatment options. The term “lupus” is also utilized when describing a handful of other dermatological conditions that are truly unrelated to lupus erythematosus, often causing confusion for rheumatologists and dermatologists alike. Sun protection and smoking cessation are central to the management of all types of CLE. Topical steroids are often the starting point for cutaneous lupus, though antimalarial agents are first line when systemic therapies are required. Should these modalities fail, a variety of other immunosuppressive medications may permit steroid-sparing while maintaining control of cutaneous lupus.

## Figures and Tables

**Table 1:** A summary of treatment options for cutaneous lesions, in order of recommendation, distinct for each diagnostic category of cutaneous lupus. Abbreviations: CLE, cutaneous lupus erythematosus; ACLE, acute cutaneous lupus erythematosus; TEN, toxic epidermal necrolysis; IVIG, intravenous immunoglobulin; SCLE, subacute cutaneous lupus erythematosus; DLE, discoid lupus erythematosus; CHLE, chilblain lupus erythematosus; LE/LP overlap, lichenoid cutaneous lupus erythematosus-lichen planus overlap syndrome.

Subset of CLE	Lineage of treatment options	Treatment of cutaneous manifestations
ACLE	First line	Sun protection Smoking cessation Topical steroids Topical calcineurin inhibitors Antimalarials
	Second line	Oral corticosteroids
TEN variant of ACLE	First line	Systemic corticosteroids
	Second line	Antimalarials IVIG Mycophenolate mofetil
SCLE	First line	Sun protection Topical steroids Topical calcineurin inhibitors Antimalarials
	Second line	Oral corticosteroids
DLE	First line	Sun protection Smoking cessation Topical steroids (fluocinonide > hydrocortisone) Topical calcineurin inhibitors Monthly intralesional triamcinolone Antimalarials
	Second line	Methotrexate Systemic retinoids Thalidomide Lenalidomide Dapsone Mycophenolate mofetil (adjuvant) Azathioprine IVIG Topical and systemic retinoids (hypertrophic DLE)
Tumid lupus	First line	Topical steroids Hydroxychloroquine, chloroquine
Lupus panniculitis	First line	Topical or intralesional steroids for overlying DLE Antimalarials Systemic corticosteroids for initial phases only
	Second line	Dapsone Mycophenolate mofetil Cyclophosphamide Thalidomide IVIG
	Third line	Rituximab
CHLE	First line	Protection from the cold Antibiotics for necrotic areas
	Second line	Topical steroids
	Third line	Systemic corticosteroids Calcium channel blockers
	Fourth line	Mycophenolate mofetil Baricitinib Ruxolitinib
LE/LP overlap	First line	Topical tacrolimus Systemic retinoids Cyclosporine
Bullous lupus	First line	Dapsone
	Second line	Systemic corticosteroids Antimalarials
	Third line	Methotrexate Azathioprine Cyclophosphamide Mycophenolate mofetil
	Fourth line	Rituximab
Neonatal lupus	First line	Sun protection Laser therapy for residual telangiectasias

## Data Availability

The data that support the findings of this study are available from the corresponding author, EEC, upon reasonable request.
